# Up-regulated expression of type II very low density lipoprotein receptor correlates with cancer metastasis and has a potential link to β-catenin in different cancers

**DOI:** 10.1186/1471-2407-10-601

**Published:** 2010-11-03

**Authors:** Lei He, Yanjun Lu, Peng Wang, Jun Zhang, Chuanchang Yin, Shen Qu

**Affiliations:** 1 Department of Biochemistry and Molecular Biology, Tongji Medical College, Huazhong University of Science and Technology, Wuhan 430030, China; 2 Clinical Laboratory, Tongji Hospital, Tongji Medical College, Huazhong University of Science and Technology, Wuhan 430030, China; 3 Department of Thoracic Surgery, Union Hospital, Tongji Medical College, Huazhong University of Science and Technology, Wuhan 430022, China; 4 Department of Breast and Thyroid Surgery, Tongji Hospital, Tongji Medical College, Huazhong University of Science and Technology, Wuhan 430030, China

## Abstract

**Background:**

Very low density lipoprotein receptor (VLDLR) has been considered as a multiple function receptor due to binding numerous ligands, causing endocytosis and regulating cellular signaling. Our group previously reported that enhanced activity of type II VLDLR (VLDLR II), one subtype of VLDLR, promotes adenocarcinoma SGC7901 cells proliferation and migration. The aim of this study is to explore the expression levels of VLDLR II in human gastric, breast and lung cancer tissues, and to investigate its relationship with clinical characteristics and β-catenin expression status.

**Methods:**

VLDLR II expression was examined using immunohistochemistry (IHC) and Western blot in tumor tissues from 213 gastric, breast and lung cancer patients, tumor adjacent noncancerous tissues by same methods. Correlations between VLDLR II and clinical features, as well as β-catenin expression status were evaluated by statistical analysis.

**Results:**

The immunohistochemical staining of VLDLR II showed statistical difference between tumor tissues and tumor adjacent noncancerous tissues in gastric, breast and lung cancers (*P *= 0.034, 0.018 and 0.043, respectively). Moreover, using Western, we found higher VLDLR II expression levels were associated with lymph node and distant metastasis in gastric and breast cancer (*P *< 0.05). Furthermore, highly significant positive correlations were found between VLDLR II and β-catenin in gastric cancer (*r *= 0.689; *P *< 0.001)breast cancer (*r *= 0.594; *P *< 0.001).

**Conclusions:**

According to the results of the current study, high VLDLR II expression is correlated with lymph node and distant metastasis in gastric and breast cancer patients, the data suggest that VLDLR II may be a clinical marker in cancers, and has a potential link with β-catenin signaling pathway. This is the first to reveal the closer relationship of VLDLR II with clinical information.

## Background

The very low density lipoprotein receptor (VLDLR) which belongs to the low density lipoprotein receptor (LDLR) family was initially cloned on the basis of its homology to the LDLR [1]. This receptor exhibits domain structures similar to those of the LDLR, except it has an extra repeat of the cysteine-rich ligand-binding domain. The tissue distribution of VLDLR is most abundantly expressed in heart, skeletal muscle and adipose tissue [2], the VLDLR is originally considered to specifically bind to VLDL and played important roles for apolipoprotein E (apoE) metabolism. Interest in VLDLR has focused mainly on its possible role in extrahepatic tissues active in fatty acid metabolism and its role as an energy source [3]. However, the physiological and pathological importance of this receptor has not been clearly identified. Previous studies has found that VLDLR is a multiple function receptor due to binding numerous ligands besides lipoproteins, including lipoprotein lipase (LPL), receptor-associated protein (RAP), thrombospondin-1, urokinase plasminogen activator/plasminogen activator inhibitor-1 complex (uPA/PAI-1) and several other proteinase-serpin complexes, causing endocytosis and affecting many cellular functions [4-8].

Furthermore, a signaling function for the VLDLR has also been recognized, as demonstrated by the ability of reelin to modulate neuronal migration, neurodevelopment, and other physiological processes in the central nervous system. Binding of reelin to VLDLR induces tyrosine phosphorylation of Disable-1 (Dab-1) in neurons [9]. In addition to neuronal migration via reelin, *in vitro *studies revealed that the VLDLR modulates cell migration via a pathway that depends on uPA [10]. That receptor-bound uPA plays a pivotal role in tumor invasion and metastasis via the generation of plasmin and subsequent degradation of the extracellular matrix in various processes like cancer cell invasion, stromal remodelling, and angiogenesis [11]. So the VLDLR appears to regulate biological processes by binding or internalization of ligands, or by transducing extracellular signals across the cell membrane.

VLDLR consists of two subtypes because of alternative splicing, namely the full-length VLDLR and type II VLDLR (VLDLR II) which lacks the O-linked sugar domain encode by the 16th exon. The tissue distribution of two VLDLR subtypes are different, the full-length VLDLR mainly expressed in heart and muscles with high lipid metabolism, whereas VLDLR II is a ~ 105 kDa protein that mainly expressed in kidney, spleen, adrenal gland and testis [12]. The previous research has also shown that VLDLR II is predominantly expressed in the gastroenterological cancer, breast cancer, lung cancer and so on [13]. Other report indicates that the O-linked sugar domain of VLDLR has been demonstrated to be responsible for cell growth inhibition, and this growth inhibition is ligand-independed [14]. These studies suggest that VLDLR II activities may be related to certain cellular functions other than its involvement in lipoprotein metabolism, and has been speculated to promote the tumor cells to proliferate and metastasis.

β-catenin is part of the cadherin-catenin complex that mediates cell-cell adhesion [15] and is a critical member of the canonical Wnt signaling pathway that is active normally in embryogenesis. It also is believed that aberrant Wnt signaling is a characteristic shared by numerous human tumors [16]. Recent studies have revealed that Wnt signal transduction plays a pivotal role in human tumor development, where it mediates the transcription of numerous downstream target genes associated with increased growth and invasion [17]. Chen *et al *reported that VLDLR is a negative regulator of the Wnt signaling pathway [18]. But in the past research, we have indicated that increased β-catenin accumulation is found in the human gastric adenocarcinoma cell line SGC7901, accompanied by an increase in VLDLR II expression. These previous results suggest that the molecular mechanism underlying the role of VLDLR II in tumor cell proliferation and migration may be related to the stability of β-catenin and the subsequent activation of the transcription of certain target genes. However, in tumor tissue levels, whether the mount of β-catenin has a correlation with VLDLR II expression need to be elucidated.

In the current study, we examined the expression of VLDLR II in different carcinoma samples by immunohistochemistry (IHC) and Western blot, and investigated whether the expression of VLDLR II are different between normal and malignant tissues. Then we further analyzed potential correlations of VLDLR II expression levels with various clinicopathologic tumor features, as well as with β-catenin expression levels.

## Methods

### Clinical cancer specimens

213 clinical specimens from gastric, breast and lung cancer patients who had undergone surgical resection (starting from 2008) at the department of surgery, both of Tongji Hospital and Union Hospital (Wuhan, China), were investigated in this study. Informed consent was obtained from patients for their tissues to be used in research. For each patient, a sample of adjacent and apparently non-affected tissue was also taken and used as normal control. The histological diagnosis of each tumor was confirmed on the hematoxylin and eosin-stained sections. Pathologic tumor, lymph node, metastasis (TNM) status were assessed in all patients according to the TNM classification system of the International Union Against Cancer/American Joint Committee on Cancer (UICC/AJCC). In order to ensure reliable results, we divided above-mentioned cancer samples into two groups, one group was comprised of 22 gastric cancers, 18 breast cancers, and 24 lung cancers, they were fixed in formalin and embedded in paraffin for IHC. Another group also consists of surgical specimens of gastric, breast, and lung cancers (52, 46, 51, respectively), and were snap-frozen immediately after resection using liquid nitrogen and stored at -80°C until used for Western blot analysis. The choice and grouping of all samples were based on blind election and random principle. All patients gave informed consent to use excess pathological specimens for research purposes. Research was carried out in compliance with Helsinki Declaration with the approval of the Ethics Committee of Tongji Medical College (Email: tongjilunli@163.com).

### Antibodies

The monoclonal antibody against VLDLR II was prepared as previously described [19], which could be applied for IHC and Western blot examination. The mouse monoclonal anti-human β-catenin was obtained from R&D systems, Minneapolis (MN, USA). The antibody against β-actin was purchased from Santa Cruz Biotechnology (CA, USA).

### Immunohistochemistry methods

Antibody staining was performed on 4-μm histological sections of formalin-fixed, paraffin-embedded tumor and adjacent normal samples. Serial 4-μm sections were mounted on pretreated glass slides, deparaffinized, rehydrated, and microwaved for 15 minutes at high power in 10 mmol/L citrate buffer (pH 6.0) to unmask the epitopes. Endogenous peroxidase was quenched using 3% H_2_O_2 _for 10 minutes; slides were then washed in phosphate-buffered saline (PBS, pH 7.5) and incubated with the 5% bovine serum albumin (BSA) for 20 minutes. Sections were incubated overnight at 4ºC with a 1:100 dilution of anti-VLDLR II antibody described above. After washing, the sections were incubated with horseradish peroxidase-conjugated secondary antibodies for 1 hour at room temperature. After washing, tissues were stained for 5 minutes with DAB (3, 3'-diaminobenzidine) chromogen and counterstained with hematoxylin, dehydrated, and coverslipped. Each experiment was performed in duplicate. Mean values from repeated counts were recorded for each case.

### Evaluation of immunohistochemistry

For each spot, areas of most intense and/or predominant staining pattern were scored by eye. Immunostaining pattern for each case was independently evaluated by two investigators (YJL and PW) at a two-headed microscope. VLDLR II expression was classified according to the following grading system: staining intensity was categorized as 0 (negative), 1 (weak), 2 (moderate), or 3 (strong). Staining areas were categorized as 0 (no positive cells), 0.1 (≤ 25% positive cells), 0.4 (> 25% and ≤ 50% positive cells), 0.6 (> 50% and ≤ 75% positive cells), or 0.9 (> 75% positive cells). To gauge both stain intensity and uniformity simultaneously, the average values for intensity for each tissue were multiplied by the average values for percentage area stained in each tissue to derive a composite histoscore *(i.e., histoscore = area × intensity*). A tissue with intense, uniform staining would be assigned the maximum histoscore of 2.7, whereas a tissue with light staining intensity (a value of 1) in only > 25% and ≤ 50% of the tissue (a value of 0.4) would get a histoscore of 0.4. Assigning a histoscore is now a commonly used method for evaluating both stain uniformity and intensity in tissues to better relate results between multiple samples from immunohistochemical studies [20].

### Western blot analysis

Prior to immunoblotting, tissues were washed three times with ice cold PBS, homogenized in lysis buffer (1% NP-40, 50 mmol/L Tris, pH 7.5, 5 mmol/L EDTA, 1% SDS, 1% sodium deoxycholate, 1% Triton X-100, 1 mmol/L PMSF, 10 mg/mL aprotinin, and 1 mg/mL leupeptin) and then incubated for 20 minutes at 4°C while rocking. Lysates were cleared by centrifugation (10 min, 12 000 rpm, 4°C). 100 μg of total protein was resolved by 10% SDS-PAGE and transferred onto nitrocellulose membranes (Immbilon, Millipore). The membranes were blocked with 5% nonfat dry milk for 2 hours at room temperature, and then incubated overnight at 4°C with first antibodies which were against VLDLR II, β-actin or β-catenin. After washing the membranes 3 times with TBST (50 mmol/L Tris-HCL pH7.6, 150 mmol/L NaCl, 0.1% Tween 20), membranes were incubated with horseradish peroxidase-conjugated secondary antibodies for 1 hour at room temperature according to the manufacturer's instructions. The membranes were washed 3 times with TBST. Detection of immunocomplexes was performed with an enhanced chemiluminescence system (NEN Life Science Products, Boston, MA), the values were normalized to β-actin expression, and the level of protein expression determined using Image Quant TL software (Amersham Pharmacia Biotech, Piscataway, USA).

### Statistical analysis

The difference of VLDLR II expression between tumor and paired normal tissues were assessed using the student *t*-test for paired values, both IHC and Western blot data were used to establish this diversities. The Mann-Whitney test or Kruskal-Wallis test (for > 2 groups) were used to analyze the association between expression levels of VLDLR II and clinical parameters. The direction and the strength of association between VLDLR II and β-catenin were evaluated and compared with the Spearman correlations test. Data were reported as mean ± SD, *P *value < 0.05 was considered statistically significant. All statistical analyses were performed with Superior Performance Software System (SPSS) 15.0 for Windows (SPSS Inc, Chicago, IL).

## Results

### Detection of cellular VLDLR II expression by IHC staining of cancer tissues

**Figure 1:**
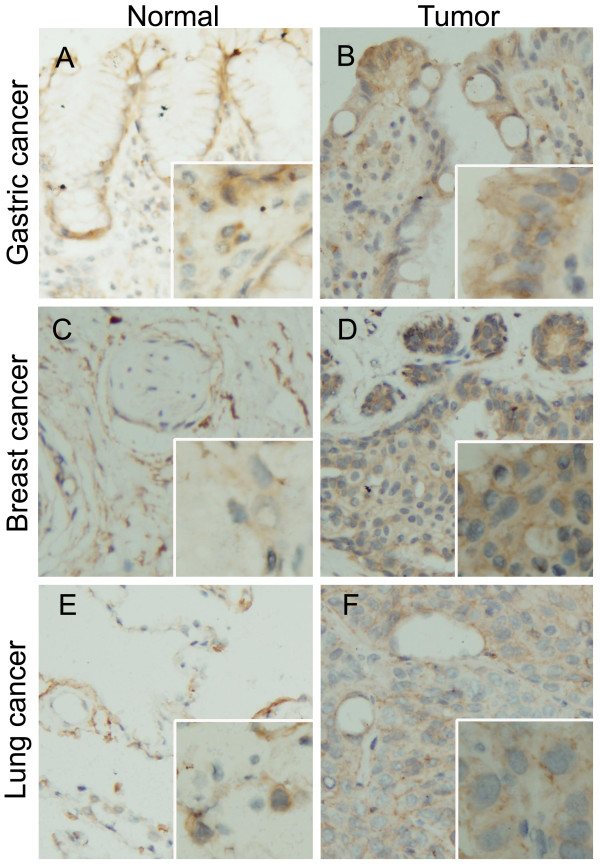
**VLDLR II detection by IHC**. Representative Immunohistochemical staining of VLDLR II protein in paraffin-embedded human different cancers and matched adjacent noncancerous tissues. A, B: Gastric normal and tumor tissue; C, D: Breast normal and tumor tissue; E, F: Lung normal and tumor tissue. VLDLR II staining show predominantly membranous localization, and a much smaller subset also showed cytoplasmic staining. Normal tissues seen in A, C, E are weak-to-moderate stained, while in cancer cells, VLDLR II staining are strongly intense (B, D, F). Original magnification: × 200; × 400 (insets).

**Figure 2:**
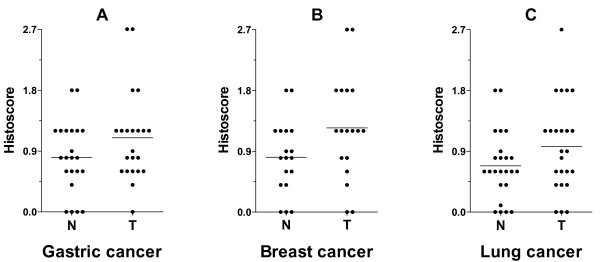
**The histoscore of VLDLR II staining in gastric (A), breast (B), lung (C) cancers and in the paired non-affected ones**. Statistical analysis with histoscore (area stained multiplied by intensity) of VLDLR II in tissues from gastric, breast and lung cancer patients (22, 18, and 24, respectively). VLDLR II expression levels are significantly higher in tumors compared with normal tissues (*P *= 0.034, 0.018, and 0.043, respectively). Abbreviations: N, matched adjacent normal tissue; T, tumor tissue.

Using IHC, we examined the distribution of VLDLR II protein in parraffin-embedded mammary tissue sections screened from gastric, breast and lung cancer patients (22, 18, and 24, respectively). VLDLR II was mainly localized in the cytomembrane of either tumor or normal cell (Figure 1). Here, the level of VLDLR II expression refers to the histoscore of cells within each tissue sample that stained positively for the special antibody, and the evaluation scoring system had been described above. The cells with brown staining were considered positively stained, and their percentage within the tissue was determined. Comparison between tumor and normal tissue revealed that VLDLR II expression levels were significantly increased in gastric, breast and lung cancer patients (*P *= 0.034, 0.018, and 0.043, respectively; Figure 2). Moreover, we observed a tissue specific profile of VLDLR II protein expression both in tumor and normal tissues, with highest levels of expression in breast cancer, followed by gastric and lung cancer. The histoscore of VLDLR II staining from different cancers were summarized in Table 1.

**Table 1:** Expression of VLDLR II in different cancers

	IHC (Histoscore of VLDLR II)				Western blot (Ratio of VLDLR II/β-actin)			
		
	N	mean	**SD**.	*P*	N	Mean	**SD**.	*P*
Gastric cancer	22				52			
Normal tissue	22	0.80	0.67	0.034*	52	0.78	0.26	0.003*
Cancer tissue	22	1.10	0.53		52	0.93	0.40	
Breast cancer	18				46			
Normal tissue	18	0.81	0.54	0.018*	46	0.93	0.43	0.001*
Cancer tissue	18	1.24	0.77		46	1.10	0.48	
Lung cancer	24				51			
Normal tissue	24	0.68	0.50	0.043*	51	0.57	0.19	0.073
Cancer tissue	24	0.97	0.67		51	0.61	0.21	

Statistical analyses were performed by Paired Students' *t *test (2-tailed).

* *P *< 0.05 is considered significant

### Expression of VLDLR II protein by Western blot in difference cancer tissues

In order to verify the IHC results and further assess VLDLR II expression whether correlates with clinical information, the cancer specimens and paired non-infected tissues from gastric, breast and lung cancer patients (52, 46 and 51, respectively) were analyzed by Western blot to quantitatively assess the proteins investigated. The values were normalized to β-actin expression. As shown in Table 1, VLDLR II was detected in cancer tissues at significantly higher levels than in the normal tissues from gastric and breast cancers (*P *= 0.003, 0.001), both results paralleled IHC data. But no significant differences were found in lung caners (*P *= 0.073).

#### VLDLR II protein in gastric cancer tissues

**Figure 3:**
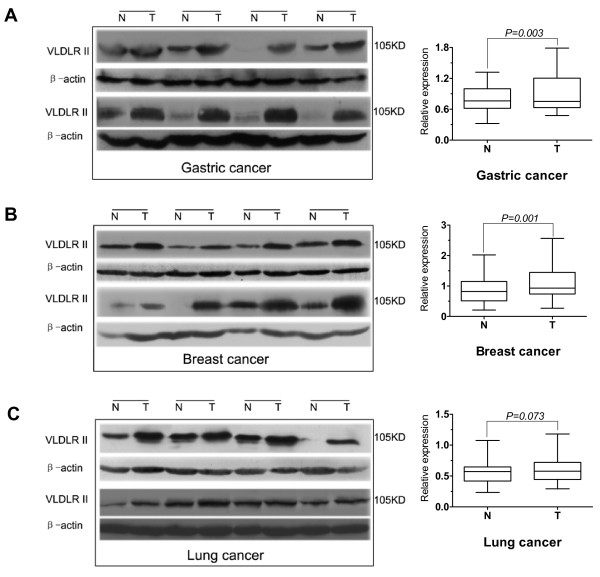
**Western blot analysis of VLDLR II expression from normal and tumor samples**. 100 μg of total protein extracted from tumor tissues and matched adjacent noncancerous tissues were tested by Western blot. A-C: Some representative samples from gastric, breast, and lung cancer patients (8 for each), indicate as N and T for normal and tumor samples. VLDLR II expression levels are higher in gastric and breast tumor tissues compared with matched adjacent noncancerous tissues (A, B). In lung tumor tissues, VLDLR II is either high or similar as compared with normal tissues (C). The same filter membranes were probed with β-actin. The bands were densitometrically scanned and referred to β-actin as internal control. The box graphs are shown the statistical analysis with relative protein expression levels of VLDLR II in tissues from gastric, breast, and lung cancer patients (52, 46, and 51, respectively). Abbreviations: N, normal tissue; T, tumor tissue.

We assessed the expression level of VLDLR II in gastric cancer tissues and matched adjacent normal tissues via Western blot analysis. Figure 3A illustrated VLDLR II expression in 8 representative gastric cancer patients. Through statistical analysis with relative protein expression levels of VLDLR II in gastric cancer tissues from 52 patients, as shown in box graph, we found that VLDLR II expression was significantly elevated in gastric cancers in comparison to the matched normal adjacent tissues, this result was consistent with which obtained via IHC. In addition, VLDLR II expression in cancer tissues was not significantly associated with age, gender, tumor size or histological grade. But elevated VLDLR II expression was associated with lymph node metastasis (*P *= 0.013) and a higher expression was noted in patients with distant metastasis (*P *= 0.004) and advanced TNM stage group (*P *= 0.011). Because VLDLR II played role for lipid metabolism, we also analysed the relationship between VLDLR II expression and other clinical features including body weight and plasma lipid profiles. The results shown that VLDLR II expression in cancer tissues was not significantly associated with body weight, total cholesterol (TC) and triglycerides (TG) (Table 2).

**Table 2:** Correlation between VLDLR II expression and clinical features in 52 gastric cancer patients

Parameters	Number	VLDLR II fold	*P*
Age at diagnosis			
≥ 60 years	26	1.34 ± 0.58	0.133
< 60 years	26	1.19 ± 0.55	
Gender			
Male	37	1.28 ± 0.58	0.709
Female	15	1.22 ± 0.55	
Body weight			
≥ 60 kg	17	1.14 ± 0.31	0.565
< 60 kg	35	1.33 ± 0.65	
TC			
> 5.20 mmol/L	11	1.11 ± 0.20	0.662
≤ 5.20 mmol/L	41	1.31 ± 0.62	
TG			
> 1.70 mmol/L	9	1.02 ± 0.23	0.204
≤ 1.70 mmol/L	43	1.32 ± 0.60	
Histological grade			
Well to moderately differentiated	30	1.19 ± 0.55	0.165
Poorly differentiated	22	1.37 ± 0.58	
Tumor size			
≥ 5 cm	25	1.40 ± 0.60	0.087
< 5 cm	27	1.14 ± 0.51	
Lymph node metastasis			
Negative	15	1.02 ± 0.51	0.013*
Positive	37	1.35 ± 0.56	
Distant metastasis			
Negative	41	1.13 ± 0.41	0.004*
Positive	11	1.78 ± 0.77	
TNM stage			
Stage I-II	22	1.08 ± 0.46	0.011*
Stage III-IV	30	1.40 ± 0.60	

Folds (VLDLR II protein in tumor/in normal) in mean ± SD, VLDLR II measured by Western blot. TC, total cholesterol (normal range: ≤ 5.20 mmol/L); TG, triglycerides (normal range: ≤ 1.70 mmol/L). Statistical analyses were performed by Mann-Whitney test.

* P < 0.05 is considered significant

#### VLDLR II protein in breast cancer tissues

We classified 46 patients with breast cancer, according to the TNM stage, histological grade, plasma lipid profiles and the levels of total VLDLR II protein in each subgroup. Significant increases in VLDLR II protein were observed in tumor group of the breast cancer patients studied (Figure 3B). As shown in Table 3, not only a global increase of VLDLR II in cancer tissues in comparison to their paired adjacent non-affected tissue, but also the higher expression of VLDLR II is associated with lymph node metastasis, distant metastasis and poorly differentiated (*P *= 0.003, 0.007 and 0.043, respectively). With regard to TNM stage, the difference is statistically significant with stage III-IV group and stag I-II group (*P *= 0.027), whereas in age, body weight, TC, TG, and tumor size, it was not.

**Table 3:** Correlation between VLDLR II expression and clinical features in 46 Breast cancer patients

Parameters	Number	VLDLR II fold	*P*
Age at diagnosis			
≥ 50 years	29	1.30 ± 0.44	0.820
< 50 years	17	1.28 ± 0.40	
Body weight			
≥ 50 kg	19	1.38 ± 0.39	0.080
< 50 kg	27	1.21 ± 0.43	
TC			
> 5.20 mmol/L	7	1.09 ± 0.34	0.178
≤ 5.20 mmol/L	39	1.31 ± 0.42	
TG			
> 1.70 mmol/L	13	1.15 ± 0.28	0.360
≤ 1.70 mmol/L	33	1.33 ± 0.45	
Histological grade			
Well to moderately differentiated	28	1.18 ± 0.36	0.043*
Poorly differentiated	18	1.43 ± 0.45	
Tumor size			
≥ 2 cm	21	1.34 ± 0.47	0.442
< 2 cm	25	1.22 ± 0.37	
Lymph node metastasis			
Negative	15	1.14 ± 0.15	0.003*
Positive	31	1.43 ± 0.45	
Distant metastasis			
Negative	32	1.16 ± 0.30	0.007*
Positive	14	1.52 ± 0.41	
TNM stage			
Stage I-II	24	1.14 ± 0.31	0.027*
Stage III-IV	22	1.41 ± 0.47	

Folds (VLDLR II protein in tumor/in normal) in mean ± SD. VLDLR II measured by Western blot. TC, total cholesterol (normal range: ≤ 5.20 mmol/L); TG, triglycerides (normal range: ≤ 1.70 mmol/L). Statistical analyses were performed by Mann-Whitney test.

* *P *< 0.05 is considered significan

#### VLDLR II protein in lung cancer tissues

In a group of 51 patients affected by lung cancer, as shown in Figure 3C, the comparison between normal and cancer tissues did not indicate significant difference in expression of VLDLR II protein, this result was different with which obtained via IHC (Table 1). However, to gain better insight into the expression level of VLDLR II in histological types of lung cancer, we analysed the difference expression of VLDLR II between normal and cancer tissues in 4 histological types of lung cancer (18 adenocarcinomas, 24 squamous carcinomas, 6 small cell lung carcinomas, and 3 adeno-squamous carcinomas). The expression of VLDLR II was significantly elevated in adenocarcinoma tissues in comparison to the corresponding adjacent non-affected tissues (*P *= 0.048), whereas in other histological types, it was not (Table 4). When analyzing expression levels of VLDLR II associated with common clinical features, we found the difference was statistically significant only in patients with distant metastasis (*P *= 0.008). Clinical variables were summarized in Table 5.

**Table 4:** Expression of VLDLR II in different histological types of lung cancer

	IHC (Histoscore of VLDLR II)				Western blot (Ratio of VLDLR II/β-actin)			
		
	N	mean	**SD**.	*P*	N	Mean	**SD**.	*P*
ADCA	10				18			
Normal tissue	10	0.71	0.12	0.034*	18	0.60	0.18	0.048*
Cancer tissue	10	1.29	0.71		18	0.70	0.25	
SQCA	13				24			
Normal tissue	13	0.68	0.68	0.651	24	0.54	0.16	0.602
Cancer tissue	13	0.75	0.58		24	0.56	0.18	
SCLC	1				6			
Normal tissue	1	0.4	--	--	6	0.56	0.20	0.554
Cancer tissue	1	0.6			6	0.59	0.30	
A-SCA					3			
Normal tissue	--	--	--	--	3	0.49	0.09	0.118
Cancer tissue	--	--			3	0.58	0.07	

ADCA indicates adenocarcinoma; SQCA, squamous cell carcinoma; SCLC, small cell carcinoma; A-SCA, adeno-squamous carcinoma. Statistical analyses were performed by Paired Students' *t *test (2-tailed).

* *P *< 0.05 is considered significant.

**Table 5:** Correlation between VLDLR II expression and clinical features in 51 Lung cancer patients

Parameters	Number	VLDLR II fold	*P*
Age at diagnosis			
≥ 55 years	30	1.16 ± 0.25	0.781
< 55 years	21	1.11 ± 0.34	
Gender			
Male	35	1.12 ± 0.31	0.424
Female	16	1.19 ± 0.37	
Body weight			
≥ 60 kg	19	1.15 ± 0.22	0.823
< 60 kg	32	1.13 ± 0.32	
TC			
> 5.20 mmol/L	12	1.03 ± 0.20	0.096
≤ 5.20 mmol/L	39	1.16 ± 0.30	
TG			
> 1.70 mmol/L	9	1.01 ± 0.35	0.166
≤ 1.70 mmol/L	42	1.17 ± 0.27	
Histological grade			
Well to moderately differentiated	38	1.11 ± 0.29	0.133
Poorly differentiated	13	1.25 ± 0.27	
Tumor size			
≥ 5 cm	22	1.20 ± 0.30	0.254
< 5 cm	29	1.09 ± 0.28	
Lymph node metastasis			
Negative	15	1.01 ± 0.37	0.090
Positive	36	1.19 ± 0.23	
Distant metastasis			
Negative	40	1.06 ± 0.26	0.008*
Positive	11	1.36 ± 0.29	
TNM stage			
Stage I-II	25	1.06 ± 0.27	0.061
Stage III-IV	26	1.25 ± 0.29	
Histological type			
adenocarcinoma	18	1.22 ± 0.29	0.190
squamous cell carcinoma	24	1.11 ± 0.31	
small cell carcinoma	6	1.03 ± 0.19	
adeno-squamous carcinoma	3	1.09 ± 0.07	

Folds (VLDLR II protein in tumor/in normal) in mean ± SD. VLDLR II measured by Western blot. TC, total cholesterol (normal range: ≤ 5.20 mmol/L); TG, triglycerides (normal range: ≤ 1.70 mmol/L). Statistical analyses were performed by Mann-Whitney test (for 2 groups) or Kruskal-Wallis test (for > 2 groups).

*P < 0.05 is considered significant.

### Correlation between VLDLR II protein and β-catenin expression levels in different cancers

**Figure 4:**
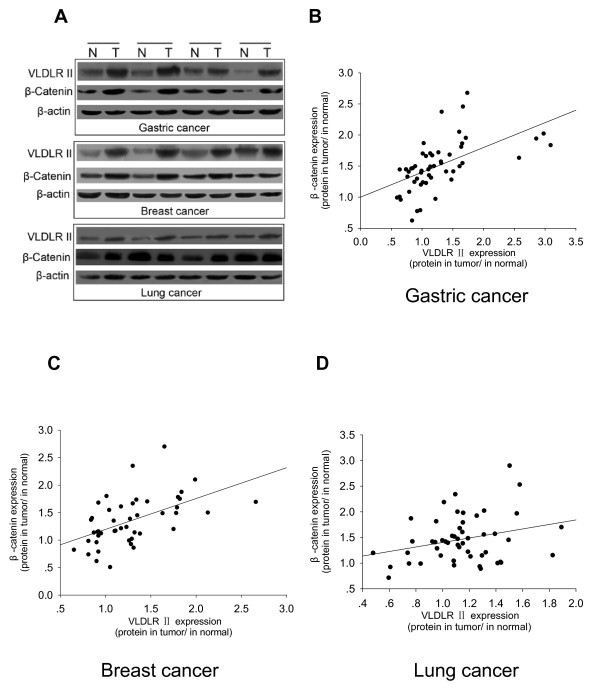
**Correlation of Western blot expression of VLDLR II with β-catenin in different cancers**. A: Representative Western blot analyses are shown of the VLDLR II and β-catenin expression in tissues from gastric, breast, and lung cancer patients (4 for each). B-D: Correlation of Western blot expression of VLDLR II with β-catenin in different cancer tissues. Calculated regression lines also are shown on the correlation plots.

In previous study, we had found that increased β-catenin accumulated in the SGC7901 cell line, accompanied by an increase in VLDLR II expression [21]. In current study, we further examined whether the VLDLR II expression could be correlated with β-catenin in tissue levels from different cancers. Except for evaluating VLDLR II expression in different cancer tissues, we also detected β-catenin in same cancer specimens and adjacent normal tissues by Western blot. β-catenin expression levels were different between normal and tumor tissues from gastric, breast, and lung cancer patients (Figure 4A). Next, we evaluated the correlation between VLDLR II and β-catenin expression, as a result, Figure 4B and 4C illustrated that highly significant positive correlations were found between VLDLR II and β-catenin in gastric cancers (Spearman test: *r *= 0.689; *P *< 0.001) and breast cancers (Spearman test: *r *= 0.594; *P *< 0.001), whereas β-catenin expression didn't correlated with VLDLR II expression in lung cancers (Spearman test: *r *= 0.222; *P *= 0.118; Figure 4D).

## Discussion

VLDLR is a member of the LDLR family [1], which includes the LDLRs, LDL receptor-related protein (LRP) and so on. These receptors have equivalent structural motifs and band many of same ligands [22]. And now, VLDLR and the other members of LDLR are known as the multifunctional receptor like "Swiss army knife" [23]. It is widely believed that the LDL receptor family participated in the tumorigenesis, which the receptor mediated endocytosis has been as a way to regulate the tumor cell migration [24]. For example, LRP mediates the endocytosis of uPAR through an indirect mechanism that depends on uPA/PAI-1 complex, and then regulates the activity of uPA/uPAR system within cancer cells [25]. Similarly, some reports suggested that VLDLR may serve as an energy source for the rapid growth of cancer cells or may function as a modulator for cancer invasion and metastasis through ligands such as uPA and its type1 inhibitor complexes [7,26]. Webb *et al*. reported that VLDLR regulates autocrine uPAR-initiated signaling and thereby regulates MCF-7 breast cancer cell motility [27].

Alternative splicing exists in many members of LDLR family, and there is some supportive evidence in which lipoprotein receptor variants may be involved in different biological processes. In the same way, VLDLR II which lacks the O-linked sugar domain encoded by the 16th exon is emerged through alternative splicing, and mainly expresses in kidney, spleen, adrenal gland and testis. However, the full-length VLDLR is most highly expressed in heart, skeletal muscle and adipose tissue with active fatty acid metabolism [12]. It was generally accepted that full-length VLDLR played an important role in the metabolism of lipoprotein enriched in apoE [1], Wada *et al*. had indicated that the O-linked sugar domain of VLDLR appeared to be responsible for the cell growth inhibition [14]. Our previous study had found that over-expression of the full-length VLDLR didn't promote SGC7901 cell migration and proliferation, while the cell migration and proliferation were increased in cells stably over-expressing VLDLR II [21]. Another report demonstrated that genetic loss of the VLDLR gene is involved in carcinogenesis including that of gastric carcinogenesis [28]. These studies suggest that VLDLR II activities may be related to certain cellular functions other than its involvement in lipoprotein metabolism.

Most results with VLDLR II had been obtained, which demonstrate pivotal role of this receptor associated with cellular proliferation and migration. Nakamura *et al*. reported that VLDLR II is the major receptor in the early phase of fetal brain, which is related to brain development [29]. Furthermore, the studies suggested that VLDLR II and apoE receptor may induce the signal transduction between extracellular Reelin and intracellular Dab1, which phosphorylates Dab1 and activates intracellular kinase to affect the migration and accurate localization of developed neuron, and VLDLR II is the important receptor binding Reelin in developed brain and inducing Reelin signal transduction pathway [30-32]. In addition, Martensen *et al*. found that VLDLR II expressed by epithelial cancer cells could function in the clearance of cell-surface-associated serine proteinase/serpin complexes in breast carcinomas [33]. We had demonstrated that VLDLR II promotes SGC7901 cell migration and invasion by regulating the expression of matrix metalloproteinase 2 (MMP2) and MMP9 [21]. Recently, we reported that uPA/PAI-1 complex can increase VLDLR expression with promoted cell proliferation and migration and stabilization of β-catenin [34].

To extend those studies, our interest is to evaluate the relevance of VLDLR II expression with clinical parameters in different human cancer tissues to characterize its role in the cancer transformation progression.

In this research, we studied the expression levels of VLDLR II in a large panel of human cancers and paired normal tissues, since it is reported that VLDLR II is expressed virtually in various cancers [13]. Our present results show that VLDLR II expression in most cancer tissues is higher than the corresponding, adjacent non-affected control tissues. Specially, VLDLR II expression in breast cancer is highest, followed by gastric and lung cancer.

Patients are also classified on the basis of the most common clinical features of each cancer. Interestingly, some of these parameters correlated with increased VLDLR II expression in cancer samples. For example, the higher VLDLR II expression is associated with lymph node metastasis, distant metastasis and advanced TNM stage in breast and gastric cancer tissues. The phenomenon showed that VLDLR II is closely related to the metastasis progression of carcinoma. In breast cancer the presence of negative prognostic markers, such as histological grade, generally correlate with higher VLDLR II expression. In lung cancer, increased VLDLR II expression also correlates with distant metastasis. These results reveal the closer relationship of VLDLR II expression with the clinicopathological guiding, up-regulated VLDLR II expression may represent an clinical marker in some cancers.

β-catenin is a key effector of the Wnt signaling pathway, which binds members of the T-cell factor (TCF)/lymphocyte enhancer binding factor (LEF) family of transcription factors and activates target genes transcription to be involved in development, tissue self-renewal and cancer [35]. It also has been reported that VLDLR can regulate the Wnt signaling, which has played an important role in tumorgenesis. Silencing of VLDLR expression by siRNA resulted in increased LRP5/6 levels and β-catenin phosphorylation in cultured cells [19]. Moreover, we have demonstrated that the β-catenin levels are associated with VLDLR II expression in the cell culture models, which causes the elevation of β-catenin levels and promotes cell proliferation and migration through the activation of the β-catenin/TCF signaling pathway [21]. In this study, we further detected the expression of β-catenin by Western blot in human cancer samples, the results show that β-catenin is higher in cancer tissues. Especially, significant positive correlations were found between VLDLR II and β-catenin in gastric and breast cancers. The data strongly suggest that there is a potential link between VLDLR II and β-catenin signaling pathway.

In addition, to strengthen the relationship between VLDLR II expression and metastasis, we have conducted a tentative experiment on the expression level of vascular endothelial growth factor (VEGF), which was regarded as a β-catenin target gene to play a important role in angiogenesis and cancer metastasis progression [36]. We found VLDLR II expression positively correlated with VEGF in breast cancer samples with distant metastasis (data not shown). We would like to carry out more extensive experiment on this topic in order to further intensify the knowledge of the relationship between VLDLR II and cancer progression.

## Conclusions

Together our findings indicate that VLDLR II may be considered as an important role in the metastasis progression of cancers, which is overexpressed in different human cancer types. To our knowledge, this is the first evidence that VLDLR II gets involved in human cancer metastasis at tissue levels. This study will help to thoroughly understand the function of VLDLR II and to open new perspectives for diagnosis and treatment of a wide range of cancers.

## Competing interests

The authors declare that they have no competing interests.

## Authors' contributions

LH designed the study, execution of most experiments, statistical analysis and drafted the manuscript. YJL participated in the design of this study, performed Western blot analysis, image analysis and helped draft the manuscript. PW performed IHC manual analysis and image analysis. JZ and CCY developed the patient database and statistical analysis. SQ proposed this study, organized the research team, interpreted all the data, and writing the manuscript. All authors read and approved the final manuscript.

## Pre-publication history

The pre-publication history for this paper can be accessed here:

http://www.biomedcentral.com/1471-2407/10/601/prepub
